# Nitrogen fixation in grasses - gluconacetobacter activates genes in sugarcane

**DOI:** 10.1186/1753-6561-8-S4-O20

**Published:** 2014-10-01

**Authors:** Thais LG Carvalho, Emília Pires, Rodrigo Saraiva, Lívia Vargas, Ana Carolina JS Bomfim, Helkin Ballesteros, José Ivo Baldani, Paulo CG Ferreira, Adriana S Hemerly

**Affiliations:** 1 Laboratório de Biologia Molecular de Plantas, Instituto de Bioquímica Médica, Centro de Ciências da Saúde, Universidade Federal do Rio de Janeiro, 21.941-590, Rio de Janeiro, RJ, Brazil; 2 Embrapa Agrobiologia, BR465, Km47, 23851-970, Seropédica, RJ, Brazil

## 

Nitrogen-fixing bacteria have been isolated from sugarcane (*Saccharum *spp.) and other grasses in an endophytic and beneficial interaction that promotes plant growth. In this interaction, bacteria colonize the intercellular spaces and vascular tissues of most plant organs without causing disease symptoms. The best characterized sugarcane endophytic diazotrophs are *Gluconacetobacter diazotrophicus*, clustered in the alpha subclass of Proteobacteria, and *Herbaspirillum seropedicae, Herbaspirullum rubrisubalbicans *and *Burkholderia *sp, clustered in the beta subclass of the Proteobacteria (Reis et al. 2000). These particular types of endophytic plant growth promoting bacteria (PGPB) can offer several benefits to host plants such as to provide nitrogen trough Biological Nitrogen Fixation (BNF) and to produce plant growth hormones (eg. auxins and gibberellins), promoting plant development, increase in biomass, defense against pathogens and tolerance to abiotic stresses [1,2].

In Brazil, BNF plays a fundamental role in sugarcane cultivation by reduction of the use of nitrogen fertilizers, making Brazilian sugarcane culture more competitive in global markets. It has been suggested that the relatively low mineral N-inputs used for Brazilian sugarcane production over the last 100 years has historically selected for varieties with a low response to applied mineral N and a high N2-fixing ability [3]. Studies on the quantification of BNF to the Brazilian sugarcane varieties, using 15N isotope dilution and 15N natural abundance, have demonstrated that the amount of fixed N2 can be highly variable. Very large BNF-inputs were observed in several sugarcane varieties, especially the wild noncommercial species Krakatau (*Saccharum spontaneum*) used in plant breeding in Brazil, as well as the commercial varieties SP 70-1143 and CB 45-3, that exhibit high yields in low-fertility soils. SP70-1143 could obtain 72% of N-requirement from BNF, while Chunee (*Saccharum barberi*), a wild noncommercial species, obtained only 14% [3,4].

An important biotechnological challenge of this century is to develop tools to apply for a sustainable agriculture, that would increase productivity using less fertilizers, pesticides, water and cultivated area. The associations that occur between sugarcane and other grasses with nitrogen-fixing endophytic bacteria have raised a large interest in their use in agriculture, in view of the positive effects on root development, and the increase in biomass and productivity. Experiments carried out at EMBRAPA Agrobiologia have shown significant results in biomass increase and grain yield due the use of diazotrophic endophytic bacteria inoculation in sugarcane, maize and rice inoculants. However, studies have also shown that the plant genotype and the environment where the association is established can influence the degree of beneficial results obtained by the plant caused by the association with endophytic bacteria.

This plant / nitrogen-fixing endophytic bacteria interaction represents a novel system of beneficial plant-microorganism association, which has unique features that remain to be characterized. The studies on BNF quantification have indicated that the selection of the best combination of endophytic diazotroph strains as well as sugarcane varieties needs to be exploited to obtain the maximum benefit from this association in agriculture. The signaling pathways by which sugarcane plants can decipher bacterial signals and respond properly for a successful association, by controlling endophyte recognition, colonization and nitrogen fixation rates, are still not clearly understood. Therefore, the major goal of our research is to develop biotechnological tools to help to obtain novel plant varieties more responsive to bacteria inoculants. The studies are based on the identification and use of plant genes that are markers and/or functionally important for the establishment of an efficient association with the endophytic diazotrophic bacteria, using genomic approaches.

We first addressed if plants were actively responding to bacteria association, mainly by studies of gene expression profiling of sugarcane plants inoculated with *G. diazotrophicus *or *H. rubrisubalbicans*. Database analysis of the sugarcane EST sequencing project (SUCEST) revealed ESTs exclusively and preferentially expressed in inoculated plants, which were functionally classified as members of several cellular and metabolic pathways [5,6]. Expression profiling analyses of sugarcane genes responsive to the endophytic diazotrophic bacteria revealed a high percentage of monocot specific genes, suggesting that the plant may have evolved special molecular mechanisms to recognize and establish efficient association with the bacteria [6]. Furthermore, several genes differentially expressed in inoculated plants do not match with any sequence of non-redundant GenBank database. These findings point the association between sugarcane and endophytic diazotrophic bacteria as a model with great potential for the discovery of new genes. Our first data indicated that sugarcane plant does not behave simply as a silent host for the growth of these bacteria and actively participates in the association with endophytic diazotrophic bacteria [5,6].

Another mechanism to be unraveled is how an endophytic and non-pathogenic type of association is established. First, sugarcane might recognize the bacteria, activating an intricate signaling network to manage plant defense responses. In a successful association, plants must allow bacteria colonization and, on the other side, should control bacteria numbers to avoid pathogenicity. We identified several members of one important class of receptor protein, the RLKs (Receptor Like Kinase), as candidates to be involved in sugarcane-nitrogen fixing bacteria association [5-8]. The data indicates that signaling pathways dependent on receptor proteins might represent an important mechanism for sugarcane recognition of endophytic diazotrophic bacteria. Also, our data suggests that the establishment of a beneficial and endophytic association would depend on the proper balance between defense responses that are induced and repressed. Ethylene is a phytohormone that often acts as a signal in pathogen defense as well as in plant development. Sugarcane EST database analysis indicated that ethylene signaling pathway is responsive to the association with diazotrophic endophytic [5,6]. Remarkably, gene expression analysis showed that some members of ethylene response pathway are repressed while others are activated after inoculation. The same genes had an opposite expression profile in response to pathogens, and were differentially regulated in sugarcane contrasting BNF genotypes [9]. Altogether, our results suggest that specific components of ethylene signaling pathway may identify a beneficial endophytic association, modulating plant response to diazotrophic endophytes.

An important question to be addressed is which mechanisms are involved in promotion of sugarcane growth during the association. Our data on sugarcane EST expression analysis showed that several members of sugarcane nitrogen assimilation apparatus and nitrogen metabolism are active in plants colonized by diazotrophic endophytes [5]. Gene expression studies on Gluthamine synthetase (GS) genes in sugarcane suggested that it could be important for the association with the endophytic diazotrophic bacteria [5]. We found that some GS genes are differential expressed between the contrasting BNF genotypes, and this may explain, at least in part, the capacity of these sugarcane genotypes to grow in low nitrogen addition [10]. We also investigated the relation of phytohormone signaling elements with the promotion of sugarcane development by the diazotrophic bacteria. Several members of phytohormone biosynthesis, transport and response pathways, including auxin and gibberellin, were preferential or exclusively expressed in inoculated plants [5,6]. Also, several genes involved in plant growth and development that are regulated by hormones were identified as preferentially expressed in sugarcane plants inoculated with the endophytic diazotrophs, such as specific genes that control cell division and expansion [5]. It indicates that some basic mechanisms controlling plant growth and development might participate in this association.

This first gene profiling analyses identified several differentially expressed sugarcane genes during early stages of an efficient association between a high BNF sugarcane genotype and the diazotrophic bacteria *G. diazotrophicus *and *H. rubrisubalbicans*. Nevertheless, the comprehension of how various sugarcane regulatory mechanisms are coordinated and connected to genotype and environmental signals, in order to control the establishment of a beneficial and endophytic type of association is still a big challenge. Currently, we are applying next generation sequencing technologies to compare expression profile in two sugarcane BNF contrasting genotypes and in response to soil conditions, such as nitrogen sources and water deficit. An integrated differential transcriptome was generated and it provided an overview of sugarcane metabolism, growth and development controlled by nitrogen, water and endophytic nitrogen-fixing bacteria during a successful association. All together, the data suggests that plant genotype, nitrogen and water soil conditions control regulatory networks that are important during the establishment of the beneficial association.

Our molecular analysis of the association is revealing sugarcane genetic controls involved in plant response to the endophytic diazotroph colonization. We propose that several levels of regulation might be operating in sugarcane during the association with endophytic diazotrophic bacteria. Signaling molecules may be involved in plant and bacterium recognition, triggering plant signaling pathways that, depending on both plant and bacterium genotypes will differentially regulate downstream mechanisms to allow bacteria colonization. To ensure an endophytic type of association, the plant must maintain a stringent control over the processes of invasion and proliferation of bacteria. To establish a beneficial association, several pathways controlling plant growth and development might be integrated with the plant response to the endophytic bacteria. The expression profiling analysis of sugarcane genes responsive to the endophytic diazotrophic bacteria allowed the identification of several pathways that might be involved in the association. Remarkably, several of them are reported to have a role both on plant defense and development, supporting a general coordination of both processes in order to control the establishment of a beneficial and endophytic type of association. Finally, the various sugarcane regulatory mechanisms might be coordinated and connected to plant genotype and environmental signals, in order to control the success of the association. We are currently searching for plant expression markers of genotypes and environmental conditions that would represent efficient associations. Further experiments are necessary to determine the function of these genes in sugarcane in order to identify pathways that might be crucial for the establishment of a successful association.
